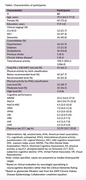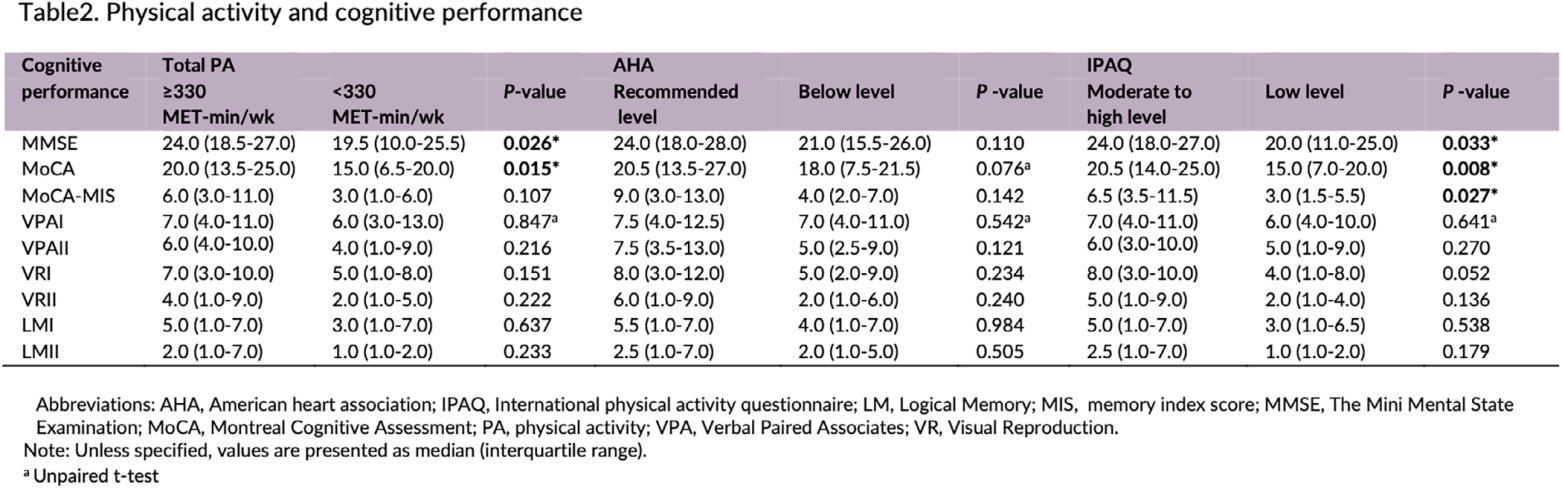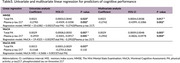# The Impact of Physical Activity on Cognitive Function in Alzheimer's Disease Diagnosed with Plasma *p*‐tau 217 in Thailand, a middle income country: A Cross‐Sectional Study

**DOI:** 10.1002/alz70858_102561

**Published:** 2025-12-26

**Authors:** Tara Rak‐areekul, Adipa Chongsuksantikul, Prawit Oangkhana, Watayuth Luechaipanit, Thanaporn Haethaisong, Thanakit Pongpitakmetha, Poosanu Thanapornsangsuth

**Affiliations:** ^1^ Division of Neurology, Department of Medicine, Faculty of Medicine, Chulalongkorn University, Bangkok, Thailand; ^2^ Memory Clinic, King Chulalongkorn Memorial Hospital, The Thai Red Cross Society, Bangkok, Thailand; ^3^ Thai Red Cross Emerging Infectious Diseases Health Science Centre, King Chulalongkorn Memorial Hospital, Bangkok, Thailand; ^4^ Thai Red Cross Emerging Infectious Diseases Health Science Centre, King Chulalongkorn Memorial Hospital, The Thai Red Cross Society, Bangkok, Thailand; ^5^ Thai Red Cross Emerging Infectious Diseases Health Science Centre, World Health Organization Collaborating Centre for Research and Training on Viral Zoonoses, King Chulalongkorn Memorial Hospital, The Thai Red Cross Society, Bangkok, Thailand; ^6^ Department of Pharmacology, Faculty of Medicine, Chulalongkorn University, Bangkok, Thailand; ^7^ Chula Neuroscience Center, King Chulalongkorn Memorial Hospital, The Thai Red Cross Society, Bangkok, Thailand

## Abstract

**Background:**

Physical activity is recognized as a non‐pharmacological approach to mitigating cognitive decline in Alzheimer's disease (AD). Prior meta‐analyses indicate that achieving at least 330 MET‐min/wk of physical activity correlates with cognitive benefits (*Dou et al., 2024*). However, the relationship between physical activity levels and cognitive performance in AD patients diagnosed via plasma *p*‐tau 217 in developing countries is not well documented. This study examines how different physical activity levels, including the 330 MET‐min/wk threshold, impact cognitive performance in this population.

**Method:**

We extracted cross‐sectional data from the ongoing INDE cohort (NCT06375213) at our hospital, including 62 participants diagnosed with AD based on plasma *p*‐tau 217 levels, ranging from cognitively unimpaired to dementia. Physical activity was measured using questionnaires, capturing total MET‐min/wk and classified according to IPAQ, AHA recommendations, and a 330 MET‐min/wk threshold. Cognitive performance was assessed with the Montreal Cognitive Assessment (MoCA), Mini‐Mental State Examination (MMSE), MoCA Memory Index Score (MoCA‐MIS), and tests of visual reproduction, logical memory, and visual paired associates. We analyzed the associations between physical activity levels and cognitive outcomes using group comparisons, Spearman correlation, and multivariate regression models, adjusting for age, sex, education, comorbidities, and *p*‐tau 217 levels.

**Result:**

Participants who achieved at least 330 MET‐min/week and those classified as having moderate to high physical activity levels on the IPAQ demonstrated significantly better cognitive performance. However, no significant differences were observed based on AHA classification. Total physical activity was positively correlated with MMSE, MoCA, and MoCA‐MIS scores. Multivariate regression analysis showed that total physical activity remained a significant predictor of MMSE (β = 0.0021, *p* = 0.017), MoCA (β = 0.0029, *p* = 0.005), and MoCA‐MIS (β = 0.0015, *p* = 0.037) scores, even after adjusting for *p*‐tau 217 levels, age, education, and comorbidities.

**Conclusion:**

Physical activity levels exceeding 330 MET‐min/wk are associated with improved cognitive performance in AD patients diagnosed via plasma *p*‐tau 217. Total physical activity positively impacted global cognitive performance, even after adjusting for confounding factors, including *p*‐tau 217 levels. Physical activities should be promoted as a modifiable intervention in clinical practice and public policy to mitigate the cognitive decline.